# Audiometric evaluation of bone conduction thresholds reveals subclinical sensorineural hearing loss in patients with ankylosing spondylitis and psoriatic arthritis

**DOI:** 10.1016/j.bjorl.2026.101813

**Published:** 2026-04-11

**Authors:** Dilara Bulut Gökten, Tolga Ersözlü, Murat Gökten, Ömer Atakan Soğur, Rıdvan Mercan

**Affiliations:** a Tekirdag Namik Kemal University, Department of Internal Medicine, Division of Rheumatology, Tekirdag, Turkey; b Tekirdag Namik Kemal University, Department of Otorhinolaryngology, Tekirdag, Turkey; c Tekirdag City Hospital, Department of Neurosurgery, Tekirdag, Turkey

**Keywords:** Ankylosing spondylitis, Psoriatic arthritis, Hearing loss, Pure tone audiometry, Bone conduction

## Abstract

**Objective:**

Hearing Loss (HL) has been increasingly recognized as a potential extra-articular manifestation in systemic autoimmune diseases, yet data on audiological involvement in Spondyloarthritis (SpA), particularly Ankylosing Spondylitis (AS) and Psoriatic Arthritis (PsA), remain limited. This study aimed to evaluate bone conduction hearing thresholds in patients with AS and PsA compared to Healthy Controls (HC) and to investigate associations with clinical and inflammatory parameters.

**Methods:**

In this retrospective cross-sectional study, Pure Tone Audiometry (PTA) was used to assess bone conduction thresholds in 53 patients with AS, 53 with PsA, and 53 age- and sex-matched HCs. Associations between hearing thresholds and clinical features, disease activity indices, comorbidities, and HLA-B27 status were analyzed.

**Results:**

Mean bone conduction thresholds were significantly higher in AS (14.85 ± 3.78 dB) and PsA (15.53 ± 9.37 dB) groups compared to controls (9.23 ± 3.42 dB, p < 0.001). In the overall cohort, hearing thresholds correlated with patient global assessment (rho = 0.307, p = 0.001), physician global assessment (rho = 0.280, p = 0.002), BASDAI (rho = 0.222, p = 0.017), ASQoL (rho = 0.304, p = 0.001), disease onset age (rho = 0.216, p = 0.020), and BMI (rho = 0.246, p = 0.008). Within disease groups, no consistent correlation was found between hearing thresholds and conventional disease activity scores.

**Conclusion:**

Subclinical sensorineural hearing impairment may be an underrecognized extra-articular feature of AS and PsA. Routine audiological assessment should be considered, especially in patients with long-standing disease, metabolic comorbidities, or subjective auditory complaints.

**Level of evidence:**

3.

## Highlights

• Bone conduction thresholds were higher in AS and PsA vs. healthy controls.
• Hearing loss may indicate subclinical cochlear involvement in SpA.
• No significant difference was found between AS and PsA thresholds.
• No consistent link between hearing thresholds and disease activity was seen.
• Routine hearing tests may be needed in SpA, even without symptoms.

## Introduction

Audiovestibular impairments, typically Sensorineural (SN), are reported in autoimmune diseases such as rheumatoid arthritis, lupus, Sjögren’s syndrome, and systemic sclerosis.^1^ Proposed mechanisms include vascular inflammation, deposition of immune complexes, hypersensitivity, autoinflammation, and ototoxic medication effects.^2^ Audiological dysfunction in Spondyloarthritis (SpA) remains relatively underexplored compared to other autoimmune diseases.

Ankylosing Spondylitis (AS), the most common form of SpA, primarily affects the axial skeleton and has a global prevalence of 0.1% to 1.4%.^3^ It can also involve peripheral joints and extra-articular systems. Reported extra-articular manifestations include anterior uveitis, cardiac conduction abnormalities, aortic regurgitation, neurological issues, apical fibrosis, and secondary renal amyloidosis.^4^ Hearing Loss (HL) has been suggested as a possible extra-articular manifestation of AS, with studies indicating a link between AS and audiovestibular impairments. While conductive HL could theoretically result from ossicular involvement, the hearing loss in AS is predominantly sensorineural, with reported prevalence ranging from 35% to 71%.^5^, ^6^, ^7^

Psoriatic Arthritis (PsA) is an inflammatory arthritis associated with psoriasis, affecting approximately 0.3%–1% of the population.^8^ It is distinguished from other forms of arthritis by unique clinical features, including Achilles tendinopathy, plantar fasciitis, and dactylitis. While PsA mainly affects joints and limits function, it may also involve extra-articular systems, including gastrointestinal, genitourinary, and cardiovascular.^8^ Recently, interest has grown in auditory complications in PsA due to links between systemic inflammation and inner ear dysfunction in autoimmune diseases. However, evidence remains limited to case reports and small cohorts.^9^

Pure Tone Audiometry (PTA) assesses hearing by measuring air and bone conduction across frequencies. Bone conduction specifically reflects cochlear and neural integrity, with elevated thresholds indicating SNHL.^10^ This study employed bone conduction measurements to assess early auditory changes and potential inner ear involvement in AS and PsA patients. Additionally, it investigated associations between bone conduction hearing thresholds and clinical features such as disease activity, systemic inflammation, extra-articular manifestations, and HLA-B27 status.

## Methods

### Study design, participants and data collection

The study was carried out retrospectively in the rheumatology and otolaryngology outpatient clinics of a tertiary care center, with data also obtained from the neurosurgery department of a collaborating hospital. It included 159 participants: 53 with AS, 53 with PsA, and 53 Healthy Controls (HC) matched for age and sex, admitted between 2021 and 2025. AS was diagnosed based on the Assessment of SpondyloArthritis International Society classification system,^11^ while PsA was diagnosed based on the Classification Criteria for Psoriatic Arthritis.^12^ HC participants had no history of autoimmune, chronic, or auditory disorders and no rheumatologic diagnoses. Exclusion criteria included age outside 18–55 years, missing clinical or laboratory data, absence of PTA results, otologic disease, conductive hearing loss (based on air-bone gap), congenital hearing loss, or neurologic conditions affecting hearing.^13^ Patients with active infection, malignancy, advanced organ failure, hearing aid use, prior ear surgery, or recent major treatment changes (≤3-months) were excluded. Informed consent was obtained from all participants.

Demographic data (age, sex, height, weight, Body Mass Index [BMI], smoking status), clinical variables (age at disease onset, symptom duration, time of diagnosis, comorbidities, current medications, and family history), and laboratory markers (C-Reactive Protein [CRP], Erythrocyte Sedimentation Rate [ESR], and Human Leukocyte Antigen B27 [HLA-B27] status) were recorded. Disease activity and functional status were evaluated using the Bath Ankylosing Spondylitis Disease Activity Index (BASDAI), Ankylosing Spondylitis Disease Activity Score with CRP (ASDAS-CRP), Ankylosing Spondylitis Quality of Life (ASQoL), patient and physician global assessments, and Visual Analog Scale (VAS) scores. Clinical assessment included major disease features: low back pain, morning stiffness, peripheral arthritis, enthesitis, dactylitis, uveitis, psoriasis, and Inflammatory Bowel Disease (IBD).

### Audiometric assessment

Otoscopy was performed in all participants prior to audiometric testing by an experienced otolaryngologist. External auditory canal and tympanic membrane were examined to exclude any signs of cerumen impaction, otitis media with effusion, tympanic membrane perforation, or other middle ear pathology. Participants with abnormal otoscopic findings were excluded from the study. Immittance audiometry, including tympanometry, was performed to assess middle ear function and to exclude ossicular chain abnormalities. Pure tone audiometry was performed by a certified audiometrist using a calibrated two-channel audiometer (Interacoustics A/S, Denmark) following international standards. Air and bone conduction thresholds were measured bilaterally at 250–8000 Hz. An air-bone gap >10 dB at any frequency indicated conductive HL, and such participants were excluded.^13^ Bone conduction thresholds (right, left, and mean) were recorded for each subject. Analyses explored associations between bone conduction hearing thresholds and demographic, clinical, inflammatory, and disease activity variables.

### Statistical analysis

Statistical analyses were performed using SPSS Statistics version 27.0. Normality of continuous variables was assessed with the Shapiro-Wilk test. Normally distributed data were expressed as mean ± Standard Deviation (SD), and non-normally distributed variables as median with Interquartile Range (IQR). Categorical variables were reported as counts and percentages. Group comparisons among the three cohorts were performed using one-way analysis of variance (ANOVA) or Kruskal-Wallis test, based on distribution. Post hoc analyses (Tukey or Dunn) were applied where relevant. Categorical data were compared using the chi-square test or Fisher’s exact test. Associations between bone conduction thresholds and demographic, clinical, or laboratory variables were assessed using Spearman’s rank correlation. Subgroup analyses (e.g., arthritis, psoriasis, dactylitis, uveitis, SpA family history) employed the Mann–Whitney *U* test or independent samples *t*-test, depending on distribution. A p-value <0.05 was considered statistically significant.

## Results

### Demographic data, clinical features, and disease activity measures

The groups were similar in terms of age, sex, height, and smoking history. A notable difference in body weight was found, with the PsA group showing the greatest mean value (p = 0.031). Regarding comorbidities, diabetes mellitus, fibromyalgia, and hyperlipidemia were significantly more common in the PsA group (p = 0.039, p = 0.016, and p = 0.014). Post hoc analyses confirmed, revealing significant differences between AS and PsA (p = 0.0126) and between PsA and controls (p = 0.0126). No meaningful variation was detected across groups regarding hypertension, fibromyalgia, or familial background in post hoc comparisons. Medication use was common in both groups: 98.1% of AS and 100% of PsA patients received treatment. Anti-Tumor Necrosis Factor (anti-TNF) agents and Nonsteroidal Anti-inflammatory Drugs (NSAIDs) were most frequently used (p < 0.001), while conventional Disease-Modifying Antirheumatic Drugs (DMARDs) like methotrexate and leflunomide were used only in PsA.

Clinical manifestations varied between patient groups. Arthritis, morning stiffness, enthesitis, and dactylitis were significantly more frequent in the PsA group (p < 0.001, p < 0.001, p = 0.002, and p < 0.001), while low back pain was reported more frequently in AS and PsA compared to controls (p = 0.008). HLA-B27 positivity was higher in the AS group (64.2%) compared to PsA (9.4%) and controls (0%) (p < 0.001). In terms of inflammatory markers and disease activity scores, CRP levels were significantly higher in both patient groups compared to controls (p = 0.004). VAS, patient and physician global scores, ASQoL, and BASDAI scores were also significantly elevated in AS and PsA groups (all p < 0.001). In post hoc analyses, no significant differences were observed between the AS and PsA groups in terms of patient global assessment (p = 1.000), physician global assessment (p = 1.000), ASQoL (p = 0.926), and BASDAI scores (p = 0.990) (see Table 1).

**Table 1:** Demographic characteristics, comorbidities, medications, laboratory parameters and disease activity indices of the study groups.

Variables	AS, n (%) or mean ± SD	PSA, n (%) or mean ± SD	HC, n (%) or mean ± SD	p
Number of cases (n)	53	53	53	1.000
Age (year)	42.96 ± 7.89	42.39 ± 6.03	40.64 ± 8.40	0.180
Sex (Male), n (%)	31 (58.5)	29 (54.7)	25 (47.2)	0.493
Height (cm)	168.5 ± 4.7	167.9 ± 5.5	168.4 ± 5.1	0.555
Weight (kg)	78.89 ± 7.07	80.38 ± 9.86	75.32 ± 11.66	***0.031***
Duration since symptom onset (years)	10.68 ± 6.78	10.47 ± 7.63	10.19 ± 5.85	0.909
Onset age (years)	31.75 ± 7.92	32.30 ± 9.09	29.47 ± 8.62	0.092
Smoking (ever)	13 (24.5)	14 (26.4)	12 (22.6)	0.903
Comorbidities, n (%)				
Diabetes mellitus	2 (3.8)	8 (15.1)	2 (3.8)	***0.039***
Hypertension	6 (11.3)	10 (18.9)	4 (7.5)	0.202
Fibromyalgia	2 (3.8)	2 (3.8)	9 (17)	***0.016***
Hyperlipidemia	1 (1.8)	7 (13.2)	1 (1.8)	***0.014***
Medications, n (%)				
Sulfasalazine	19 (35.8)	14 (26.4)	0 (0)	***<0.001***
NSAIDs	35 (66)	25 (47.1)	0 (0)	***<0.001***
Anti- TNF biologics	45 (84.9)	38 (71.6)	0 (0)	***<0.001***
JAK inhibitors	1 (1.8)	1 (1.8)	0 (0)	0.603
IL-17 inhibitors	4 (7.5)	7 (13.2)	0 (0)	***0.027***
Duloxetine	2 (3.6)	2 (3.6)	4 (7.2)	0.442
Methotrexate	0 (0)	30 (56.6)	0 (0)	NA
Leflunomide	0 (0)	15 (28.3)	0 (0)	NA
Cyclosporine	0 (0)	2 (3.6)	0 (0)	NA
Ixekizumab	0 (0)	2 (3.6)	0 (0)	NA
Clinical manifestations, n (%)				
Low back pain	30 (56.6)	27 (50.9)	15 (28.3)	***0.008***
Arthritis	8 (15.1)	36 (67.9)	0 (0)	***<0.001***
Crohn’s disease	2 (3.8)	5 (9.4)	0 (0)	0.077
Morning stiffness	21 (39.6)	29 (54.7)	4 (7.5)	***<0.001***
Psoriasis	0 (0)	53 (100)	0 (0)	NA
Enthesitis	12 (22.6)	23 (43.4)	6 (11.3)	***0.002***
Uveitis	3 (5.7)	3 (5.7)	0 (0)	0.210
Dactylitis	1 (1.9)	10 (18.9)	0 (0)	***<0.001***
Family history	21 (39.6)	18 (34)	8 (15.1)	***0.007***
Ulcerative colitis	0 (0)	2 (3.8)	0 (0)	NA
HLA-B27 positivity	34 (64.2)	5 (9.4)	0 (0)	***<0.001***
Laboratory parameters and disease activity indices (mean ± SD or median [IQR])				
ASDAS-CRP	0.98 ± 0.67	0.95 ± 0.59	NA	NA
ESR	8.0 [5.0–13.0]	10.0 [6.0–14.0]	7.0 [5.0–11.0]	0.253
CRP	8.51 [1.90–10.84]	7.00 [2.40–9.00]	4.00 [2.10–6.09]	***0.004***
VAS scores	2.02 ± 1.90	2.58 ± 2.17	1.23 ± 1.69	***0.002***
Patient global scores	2.0 [1.0–5.0]	2.0 [1.0–5.0]	1.0 [0.0–2.0]	***<0.001***
Doctor global scores	1.0 [0.0–2.0]	1.0 [1.0–2.0]	0.0 [0.0–1.0]	***<0.001***
ASQoL scores	6.81 ± 3.39	7.06 ± 3.62	3.43 ± 3.11	***<0.001***
BASDAI	2.95 ± 1.66	3.00 ± 1.46	1.69 ± 1.58	***<0.001***

AS, Ankylosing Spondylitis; PsA, Psoriatic Arthritis; HC, Healthy Controls; SD, Standard Deviation; NSAIDs, Non-Steroidal Anti-Inflammatory Drugs; JAK, Janus kinase; IL, Interleukin; ASDAS-CRP, Ankylosing Spondylitis Disease Activity Score using C-Reactive Protein; ESR, Erythrocyte Sedimentation Rate; CRP, C-Reactive Protein; VAS, Visual Analogue Scale; ASQoL, Ankylosing Spondylitis Quality of Life questionnaire; BASDAI, Bath Ankylosing Spondylitis Disease Activity Index.

### Bone conduction thresholds across groups

**Fig. 1:**
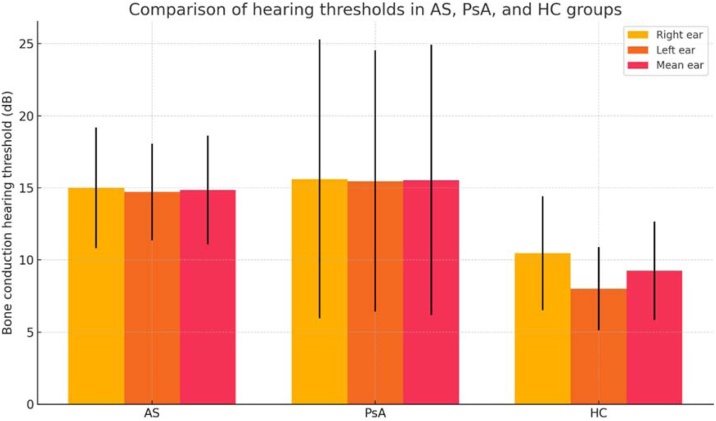
Elevated hearing thresholds in anyklosing spondylitis and psoriatic arthritis compared to healthy controls. AS, Anyklosing Spondylitis; PsA, Psoriatic Arthritis; HC, Healthy Controls.

Tympanometric evaluation demonstrated normal middle ear function (type A tympanograms) in all participants included in the final analysis, with no evidence of middle ear effusion or ossicular chain dysfunction. To minimize the potential influence of middle ear pathology and to more accurately reflect cochlear function, only bone conduction thresholds were included in the analysis. In the overall group, the mean air-bone gap was 5.31 ± 3.31 dB. Bone conduction thresholds assessed by PTA differed significantly between groups. AS and PsA patients showed higher bone conduction thresholds than HC in all parameters. Right ear means were 14.99 ± 4.19 dB (AS), 15.61 ± 9.67 dB (PsA), and 10.45 ± 3.96 dB (HC) (p < 0.001). Left ear thresholds were also higher in AS (14.71 ± 3.36 dB) and PsA (15.45 ± 9.06 dB) than in controls (8.00 ± 2.88 dB) (p < 0.001). Mean bone conduction values (average of both ears) were elevated in AS (14.85 ± 3.78 dB) and PsA (15.53 ± 9.37 dB) compared to controls (9.23 ± 3.42 dB) (p < 0.001). Given the higher variability observed particularly in the PsA group, bone conduction thresholds are additionally presented as median and interquartile range. Non-parametric comparisons yielded results consistent with the primary analyses. Post hoc tests showed significantly higher thresholds in AS and PsA compared to controls for both ears (right: AS vs. HC p = 0.041; PsA vs. HC p = 0.022; left: both p < 0.001). No significant differences were found between AS and PsA in any comparison (right: p = 0.964; left: p = 0.832; mean: p = 0.366) (see Table 2 and Fig. 1).

**Table 2:** Comparison of bone conduction hearing thresholds (right, left, and mean) in pure tone audiometry among ankylosing spondylitis, psoriatic arthritis, and healthy control groups.

Bone conduction hearing thresholds (dB)	AS, mean ± SD	PSA, mean ± SD	HC, mean ± SD	p
Right ear	14.99 ± 4.19	15.61 ± 9.67	10.45 ± 3.96	**<0.001**
Left ear	14.71 ± 3.36	15.45 ± 9.06	8.00 ± 2.88	**<0.001**
Mean	14.85 ± 3.78	15.53 ± 9.37	9.23 ± 3.42	**<0.001**
Median (IQR)^a^	13.51 (11.39–15.00)	13.66 (8.60–20.58)	9.00 (6.55–11.10)	**<0.001**

AS, Ankylosing Spondylitis; PsA, Psoriatic Arthritis; HC, Healthy Controls; SD, Standard Deviation; dB, Decibel, IQR, Interquartile Range.

a Median values were compared using the Kruskal-Wallis test.

### Correlation analysis involving disease activity scores, clinical parameters, and demographic data

Higher mean bone conduction thresholds were significantly correlated with worse patient-reported outcomes and disease activity. In the overall cohort, thresholds correlated with ASQoL (rho = 0.208, p = 0.008), BASDAI (rho = 0.319, p < 0.001), patient global (rho = 0.317, p < 0.001), and physician global scores (rho = 0.289, p < 0.001). In PsA, significant correlations were observed with both patient (rho = 0.288, p = 0.036) and physician global assessments (rho = 0.287, p = 0.037).

HLA-B27 positivity was linked to higher bone conduction hearing thresholds in the total cohort (rho = 0.271, p < 0.001), but not within subgroups. Patients with clinical arthritis had higher mean thresholds than those without (16.29 ± 5.17 dB vs. 11.59 ± 5.09 dB, p = 0.0043). Dactylitis was associated with elevated right ear thresholds (18.54 ± 6.70 dB vs. 11.71 ± 6.80 dB, p = 0.0236), though the difference in mean thresholds approached but did not reach significance (p = 0.0927). Psoriasis showed a weak positive correlation with mean thresholds (rho = 0.164, p = 0.039), and affected individuals had significantly higher average values (13.58 ± 7.34 dB vs. 11.43 ± 4.29 dB, p = 0.039). Onset age correlated with higher mean thresholds in the total group (rho = 0.156, p = 0.049) and remained significant in PsA (rho = 0.312, p = 0.022). In AS, onset age was associated with left ear thresholds (rho = 0.359, p = 0.008). In PsA, longer disease duration showed a weak but significant correlation with worse left ear thresholds (rho = −0.280, p = 0.042). BMI correlated positively with left ear thresholds in the total group (rho = 0.216, p = 0.006) and in PsA (rho = 0.326, p = 0.017).

No significant correlations were observed between bone conduction thresholds and CRP, ESR, ASDAS-CRP, family history, smoking, back pain, or morning stiffness in the overall cohort or subgroups (see Table 3).

**Table 3:** Spearman correlation between disease activity scores, clinical, laboratory, and audiometric parameters among study groups.

Mean ear bone conduction treshold	All group (Spearman rho, p-value)	AS (Spearman rho, p-value)	PsA (Spearman rho, p-value)	HC (Spearman rho, p-value)
Disease activity indices				
ASQoL	0.208, ***0.008***	0.028, 0.840	−0.045, 0.765	−0.169, 0.275
BASDAI	0.319, ***<0.001***	−0.038, 0.786	0.255, 0.066	0.116, 0.445
Patient global score	0.317, ***<0.001***	0.256, 0.064	0.288, ***0.036***	0.182, 0.193
Doctor global score	0.289, ***<0.001***	0.249, 0.076	0.287, ***0.037***	0.152, 0.287
CRP	−0.024, 0.760	−0.036, 0.798	0.090, 0.520	−0.059, 0.676
ESR	0.058, 0.468	0.013, 0.926	−0.053, 0.708	−0.023, 0.871
ASDAS-CRP	0.099, 0.214	−0.154, 0.271	0.154, 0.269	−0.010, 0.942
Clinical parameters				
HLA-B27 positivity	0.271, ***<0.001***	0.138, 0.325	0.095, 0.500	NA
Arthritis	0.227, ***0.004***	0.161, 0.252	0.179, 0.204	NA
Family history	0.087, 0.274	0.015, 0.921	−0.025, 0.871	0.329, 0.166
Uveitis	0.079, 0.319	0.104, 0.464	0.005, 0.985	NA
Dactylitis	0.314, 0.091	NA	0.015, 0.612	NA
Enthesitis	0.096, 0.228	0.166, 0.237	0.015, 0.921	NA
Psoriasis	0.164, ***0.039***	0.126, 0.372	−0.069, 0.623	NA
Morning stiffness	0.099, 0.213	0.078, 0.579	−0.109, 0.437	NA
Crohn’s disease	0.132, 0.100	NA	0.209, 0.150	NA
Smoking (ever)	−0.023, 0.773	0.195, 0.162	−0.087, 0.538	−0.103, 0.463
Low back pain	0.051, 0.521	0.109, 0.438	−0.106, 0.449	−0.096, 0.495
Disease duration	−0.094, 0.237	−0.001, 0.994	−0.231, 0.096	NA
Onset age	0.156, ***0.049***	0.010, 0.940	0.312, 0.022	NA
BMI	0.107, 0.180	−0.004, 0.978	0.189, 0.176	−0.033, 0.816

AS, Ankylosing Spondylitis; PsA, Psoriatic Arthritis; HC, Healthy Controls; ASQoL, Ankylosing Spondylitis Quality of Life Questionnaire; BASDAI, Bath Ankylosing Spondylitis Disease Activity Index; VAS, Visual Analog Scale; ESR, Erythrocyte Sedimentation Rate; CRP, C-Reactive Protein; HLA-B27, Human Leukocyte Antigen B27; BMI, Body Mass Index; NA, Not Applicable.

## Discussion

A key strength of this study is its dual focus on AS and PsA, enabling direct comparison of auditory involvement between these SpA subtypes. To our knowledge, it is the first to systematically assess bone conduction thresholds in both groups using PTA. This approach offers objective evidence of subclinical hearing loss in SpA.

The first HL case in AS was reported by Magaro et al. İn 1984^14^ as middle ear arthritis with mainly conductive loss. However, later reports suggest that SNHL is more common in AS.^7^^,^^15^ The characteristic auditory profile of AS remains debated. In this study, bone conduction thresholds were significantly higher in AS compared to HC, indicating impaired hearing function. Similar auditory impairment in AS has been reported by Casellini and Alatas et al., supporting the present findings.^7^^,^^16^ Various mechanisms have been proposed for HL in AS, including immune-mediated vasculitis, stria vascularis involvement, inner ear autoantibodies, ototoxic drugs, and cochleovestibular damage from secondary amyloidosis.^7^^,^^17^ Savastano et al. linked sulfasalazine’s active metabolite, salicylate, to HL, with cochlear effects persisting post-discontinuation.^17^ Ajmani et al. highlighted NSAID ototoxicity and suggested a dose-dependent relationship with HL.^18^ In this study, 98.1% of AS patients were on treatment. Besides AS-related cochlear involvement, medication-related ototoxicity may have contributed to the elevated hearing thresholds compared to controls.

Studies in the literature have reported conflicting results regarding the association between disease activity indices and HL. Koç et al. reported no association with clinical or laboratory variables, whereas Amor-Dorado et al. identified links with HLA-B27 positivity, uveitis, and hip involvement.^5^^,^^19^ In a recent review by Flora Yan et al., longer disease duration was highlighted as a potential risk factor for HL in AS patients.^20^ Although hearing thresholds were associated with variables like patient/physician global scores, ASQoL, disease onset, BASDAI, and arthritis in the overall group, no significant correlations were found within the AS group alone. As seen with uveitis, HL may represent an extra-articular feature with limited linkage to standard disease activity indices.^21^ These associations in the overall group may reflect differences between patient and control groups, rather than within the AS group itself. The lack of correlation in AS may stem from homogeneity in disease activity or hearing thresholds, or suggest that HL is more influenced by systemic or treatment-related factors than by disease activity alone.

Data on HL in PsA are limited, mostly from case series and single-center studies.^22^ Consistent with Akdağ et al., who reported cochlear abnormalities in asymptomatic PsA patients via PTA, the current study also found elevated bone conduction thresholds in this group.^9^ Although Akdağ et al. found no link between disease severity and HL, the current study similarly showed no significant correlation between bone conduction thresholds and disease activity indices, except for patient and physician global assessments. Likewise, Semenov et al. reported that PsA was independently associated with both self-reported and audiometrically confirmed HL. They observed significantly higher bone conduction thresholds in PsA patients compared to controls, supporting a possible link to sensorineural HL. HL also partially mediated the relationship between PsA and psychiatric comorbidities such as depression.^23^ However, psychiatric parameters were not evaluated in this study, so the potential mediating role of HL in psychological outcomes could not be assessed.

Several mechanisms have been proposed to explain the link between PsA and HL. Chronic systemic inflammation may impair cochlear microcirculation and damage outer hair cells, while proinflammatory cytokines can reduce cochlear blood flow.^24^ Autoimmune responses targeting inner ear antigens have also been implicated in SNHL. Supporting this, Güneş et al. found higher HL prevalence in 39 PsA patients without auditory symptoms compared to controls.^24^ However, findings remain inconsistent, as Karabulut et al. reported no cochlear damage in patients with psoriasis.^25^ Güneş et al. suggested these discrepancies may reflect distinct auditory profiles in psoriasis and PsA. Similarly, this study found a correlation between psoriasis and elevated thresholds, suggesting a possible shared inflammatory mechanism. The precise biological basis of this association remains unclear.

In recent years, objective measures of cochlear and retrocochlear function particularly Otoacoustic Emissions (OAE) and Auditory Brainstem Response (ABR) have been increasingly emphasized for detecting subtle, subclinical auditory involvement in immune-mediated diseases. For example, a 2023 study in psoriatic disease assessed transient-evoked OAE (with contralateral suppression) alongside audiometry and reported altered OAE parameters, supporting the possibility of early outer hair cell dysfunction even when conventional thresholds remain within normal limits.^26^ In parallel, contemporary reviews in autoimmune conditions highlight that extending the audiological test battery to include ABR and OAE may reveal early pathway abnormalities that PTA alone can miss.^27^ Moreover, inner-ear involvement is biologically plausible in chronic systemic inflammation, where cytokine-driven microvascular and inflammatory mechanisms have been implicated; population data also suggest that systemic inflammation indices correlate with HL risk.^28^ Accordingly, future studies in AS and PsA could incorporate OAE/ABR together with inflammatory biomarkers to better characterize cochlear inflammation and clarify whether the observed differences represent early cochlear involvement.

Several limitations should be considered. The retrospective design introduces selection bias and limits causal interpretation. Small subgroup sizes may have reduced power to detect subtle associations, especially within individual disease groups. Although mean bone conduction thresholds in all groups remained within the audiologically normal range, the statistically significant differences observed between patients with AS, PsA, and HCs may reflect subclinical SNHL. This finding suggests early cochlear involvement that is not yet perceived by patients and may precede clinically overt hearing impairment. However, as hearing thresholds did not exceed 20 dB, the immediate clinical impact of these changes remains uncertain. More sensitive objective auditory assessments, such as OAE, ABR or electrocochleography, may provide further insight into early cochlear or neural dysfunction and should be considered in future studies to better characterize subclinical auditory involvement in SpA. Additionally, psychiatric comorbidities and medication dosages were not systematically evaluated. Differences in comorbidity profiles and medication exposure, particularly in the PsA group, may have influenced audiometric outcomes and represent potential confounding factors that were not fully controlled for in the present analysis. Given the exploratory nature of the study and the relatively limited sample size, robust multivariable adjustment to control for multiple potential confounders was not feasible.

Despite these limitations, the study benefits from a well-matched control group, strict exclusion of conductive hearing loss and presbycusis, and detailed subgroup analyses by disease activity, comorbidities, and HLA-B27 status. Assessing clinical, inflammatory, and demographic correlates of bone conduction hearing thresholds adds depth to understanding subclinical HL in SpA. Future research should focus on prospective studies with larger cohorts.

## Conclusion

This study showed that patients with AS and PsA have significantly higher bone conduction thresholds than HC, indicating subclinical SNHL. Although no consistent links were found between disease activity indices and bone conduction hearing thresholds within groups, factors like global assessments, age at onset, BMI, and comorbidities, particularly in PsA, may impact auditory function. These results support considering HL as a potential extra-articular feature of SpA. Routine audiologic evaluation may be beneficial, especially in patients with long disease duration or metabolic risk factors.

## ORCID ID

Ömer Atakan Soğur: 0009-0001-2817-2088

Rıdvan Mercan: 0000-0003-1537-2192

## Ethical approval

Ethical approval for the study was obtained from the institutional review board (approval date: 28.01.2025; reference number: 2025.18.01.18), and all procedures were performed in line with the principles outlined in the Declaration of Helsinki.

## Funding

No specific funding was received for this study.

## Data availability statement

The data that support the findings of this study are available from the corresponding author upon reasonable request.

## Declaration of competing interest

The authors declare no conflicts of interest.

## References

[1] Rahne T., Clauß F., Plontke S.K., Keyßer G. (2017). Prevalence of hearing impairment in patients with rheumatoid arthritis, granulomatosis with polyangiitis (GPA, Wegener’s granulomatosis), or systemic lupus erythematosus. Clin Rheumatol..

[2] Andrade S.O., Appenzeller S. (2022). Ear, nose and throat manifestations of autoimmune and autoinflammatory diseases: a rheumatology perspective. Braz J Otorhinolaryngol..

[3] Dean L.E., Jones G.T., MacDonald A.G., Downham C., Sturrock R.D., Macfarlane G.J. (2014). Global prevalence of ankylosing spondylitis. Rheumatology (Oxford)..

[4] Siao W.-Z., Liu C.-H., Wang Y.-H., Wei J.C.-C., Jong G.-P. (2021). Increased risk of valvular heart disease in patients with ankylosing spondylitis: a nationwide population-based longitudinal cohort study. Ther Adv Musculoskelet Dis..

[5] Amor-Dorado J.C., Barreira-Fernandez M.P., Vazquez-Rodriguez T.R. (2011). Audiovestibular manifestations in patients with ankylosing spondylitis. Medicine (Baltimore)..

[6] Dagli M., Sivas Acar F., Karabulut H., Eryilmaz A., Erkol Inal E. (2007). Evaluation of hearing and cochlear function by DPOAE and audiometric tests in patients with ankylosing spondilitis. Rheumatol Int..

[7] Alatas N., Yazgan P., Oztürk A., San I., Iynen I. (2005). Audiological findings in patients with ankylosing spondylitis. J Laryngol Otol..

[8] Gladman D.D., Antoni C., Mease P., Clegg D.O., Nash P. (2005). Psoriatic arthritis: epidemiology, clinical features, course, and outcome. Ann Rheum Dis..

[9] Akdag M., Uçmak D., Özkurt F.E., Bozkurt M., Akkurt Z.M., Topçu İ. (2015). Evaluation of Hearing and Outer Hair Cell Function of Cochlea in Patients With Psoriatic Arthritis. Clin Exp Otorhinolaryngol..

[10] Brandt J.P., Winters R. (2023).

[11] Rudwaleit M., van der Heijde D., Landewé R. (2009). The development of Assessment of SpondyloArthritis international Society classification criteria for axial spondyloarthritis (part II): validation and final selection. Ann Rheum Dis..

[12] Taylor W., Gladman D., Helliwell P., Marchesoni A., Mease P., Mielants H. (2006). Classification criteria for psoriatic arthritis: development of new criteria from a large international study. Arthritis Rheum..

[13] Morse-Fortier C., Doney E., Fallon K., Remenschneider A. (2024). Audiometric evaluation and diagnosis of conductive hearing loss. Operative Tech Otolaryngol Head Neck Surg..

[14] Magaro M., Ceresia G., Frustaci A. (1984). Arthritis of the middle ear in ankylosing spondylitis. Ann Rheum Dis..

[15] Erbek S.S., Erbek H.S., Yilmaz S., Topal O., Yucel E., Ozluoglu L.N. (2006). Cochleovestibular dysfunction in ankylosing spondylitis. Audiol Neurotol..

[16] Casellini C., Citera G., Rosemffet M., Ruggeri S., Saviotti A., Cocco J.A.M. (2005). Audiovestibular disorders in patients with ankylosing spondylitis. J Clin Rheumatol..

[17] Savastano M., Gino M., Gloria B., Punzi L. (2005). Tinnitus and bilateral sensorineural hearing loss: Ankylosing spondylitis or a side-effect of sulphasalazine treatment?. Acta Otolaryngol..

[18] Ajmani S., Keshri A., Srivastava R., Aggarwal A., Lawrence A. (2019). Hearing loss in ankylosing spondylitis. Int J Rheumatic Dis..

[19] Koç A., Emre İE. (2015). Audiovestibular manifestations in patients with ankylosing spondylitis ‒ A case report and review of the literature. J Int Adv Otol..

[20] Yan F., Reddy P.D., Nguyen S.A., Ward C., Meyer T.A. (2021). Hearing loss in patients with ankylosing spondylitis: a systematic review and metaanalysis. J Rheumatol..

[21] Stolwijk C., van Tubergen A., Castillo-Ortiz J.D., Boonen A. (2015). Prevalence of extra-articular manifestations in patients with ankylosing spondylitis: a systematic review and meta-analysis. Ann Rheum Dis..

[22] Coulton A.K., Gubbins W.R., Young-Min S. (2023). E016 Erosive psoriatic arthritis causing deafness. Rheumatology..

[23] Semenov Y.R., Hsiang E.Y., Huang A. (2019). Association between psoriasis with arthritis and hearing impairment in US adults: data from the national health and nutrition examination survey. J Rheumatol..

[24] Gunes A., Gundogdu I., Mutlu M., Ozturk E.A., Cakci A., Akin I. (2016). Functions of the inner ear in psoriatic arthritis. Auris Nasus Larynx..

[25] Karabulut H., Karadag A.S., Dagli M. (2010). Investigation of Hearing and Outer Hair Cell Function of Cochlea in Patients with Psoriasis. J Int Adv Otol..

[26] Parabakan Polat A., Erbek H.S. (2023). Evaluation of auditory functions in patients with psoriasis. Indian J Otolaryngol Head Neck Surg..

[27] Sieśkiewicz M., Rębacz D., Sieśkiewicz A. (2024). Hearing impairment in systemic sclerosis patients-what do we really know?. Front Med (Lausanne)..

[28] Zhou T., Mao J., Zhu P., Yu X., Yang X. (2024). Association between the systemic immuno-inflammation index and hearing loss: result from NHANES 2009-2018. Front Neurol..

